# Supplementary material for: Identification of an analgesic lipopeptide produced by the probiotic Escherichia coli strain Nissle 1917
Source: Nat Commun. 2017 Nov 3;8:1314. doi: 10.1038/s41467-017-01403-9 (PMC5670229; doi:10.1038/s41467-017-01403-9)
Supplement: Supplementary file 1 — Supplementary Information [file 41467_2017_1403_MOESM1_ESM.pdf]

Supplementary Note 1: Interpretation of the proposed mechanisms for the main dissociative pathways of the activated lipoamino acid ions.

In this study, dissociations of deprotonated N-acyl aminoacids and N-acyl dipeptides formed in electrospray are investigated under high resolution MS/MS conditions to get elemental composition of both the precursor and product ions, information useful for interpretation of fragmentations. Mechanistic interpretation of these product ions generated by precursor ion activation under low collision energy conditions processes is based on mechanisms used in different studies reported in a recent review[1], and used for dissociations of lipoamino acids[2]. In the higher-energy collision dissociation (i.e., HCD mode as a non-resonant process), charge promotes ion dissociation through competitive and consecutive dissociation pathways in collision cell. Various concepts were used in our interpretation of fragmentations and we considered that : (i) the molecular deprotonation in electrospray may take place in competition at the various acidic sites (i.e., herein at carboxylic acid, amide, peptide bond as well as at enolizable methylene group in α position of the C=O site), (ii) stabilization of charge in molecular species can take place by internal charge solvation by conformation folding, and (iii) prior to dissociation, the negative ions may isomerize into ion-neutral intermediate able to dissociate either directly or after internal proton transfer between partners of complex.

1. Dissociation of the deprotonated C14Asn*OH* (fig. 1C) and C12Asn*OH* (fig. S1B)

From the deprotonated enolate molecular form of C14Asn*OH* and C12Asn*OH* (i.e., m/z 341 and m/z 313, respectively), different cleavages occur yielding:

(a) m/z 323 (fig. 1C) and m/z 295 (Supplementary Fig. 1B) provided by loss of water. This common loss takes place by a stepwise process. It involves a first isomerization of the deprotonated molecule (within the amidate form) into reactive tetrahedral intermediate formed through a nucleophilic attack at the carboxylic acid group through Asn side chain folding (Supplementary Fig. 2a). This intermediate may consecutively isomerizes into an ion-neutral complex (ID_1) constituted

by a bis-substituted and unsaturated γ-lactone and the OH^- reagent. This latter may remove proton from one acid site of the neutral partner (Supplementary Fig. 2a) allowing the water loss. Stabilized by the charge delocalization, this ion seems to do not further dissociate.

(b) m/z 252 (fig. 1C) and m/z 224 (Supplementary Fig. 1b). Fast consecutive dissociations through the loss of the isocyanic acid giving rise to formation of a very reactive $[(M\text{-}H)\text{-}OCNH]^-$ ion. This one transposes promptly into the ID_2 ion-dipole complex (Supplementary Fig. 2b), from which, the neutral partner i.e., the N-acyl imine neutral, transfers proton to the formulate anion. By the formic acid loss, the vinyl amidate anion are formed at m/z 252 and m/z 224 as displayed in the HCD spectra of m/z 341 (Fig. 2C) and m/z 313 (Supplementary Fig. 1B).

(c) m/z 226 (fig. 1C) and m/z 198 (Supplementary Fig. 1B) provided *via* formation of ion-neutral intermediate (e.i., ID_3, Supplementary Fig.2c). This latter can dissociate directly to give rise to formation of deprotonated fatty amides at m/z 226 and m/z 198 for C14Asn*OH* and C12AsnOH, respectively. Competitively to the direct cleavage, intra partner proton transfer from maleamic acid to the deprotonated fatty acid amide can take place yielding, after ion-neutral dissociation, the common deprotonated maleamic acid (m/z 114), characteristic to the Asn moiety. Consecutively, the m/z 114 product ion may lose water *via* ion-neutral ID_4 intermediate (Supplementary Fig. 2c).

(d) and finally, the common m/z 131 product ion corresponding to the $[Asn\text{-}H]^-$ ion. This fragment ion is directly (Supplementary Fig. 2d) generated from the [C14Asn*OH*-H]$^-$ and [C12Asn*OH*-H]$^-$ precursor ion which are deprotonated at the carboxylic acid site. This contrasts with the precursor ions yielding the previous product ion series. Indeed, in these former cases, the deprotonated molecule carried the charge at the amide site.

2. Dissociation of the deprotonated C12AsnGaba*OH* (Fig. 3C), C12AsnLeu*OH* (Supplementary Fig. 1c) and C14AsnLeu*OH* (Supplementary Fig. 3B)

The HCD spectra of the [C12AsnGaba*OH*-H]$^-$ (m/z 398), [C12AsnLeu*OH*-H]$^-$ (m/z 426) and [C14AsnLeu*OH*-H]$^-$ (m/z 454) precursor ions, deprotonated at the amide side chain group, display

several series of product ions formed according to similar fragmentation pathways. These ones involve competitive dissociations of ion-neutral intermediates (generated by precursor ion isomerization) to yield the following complementary ion pairs:

(a) m/z 295//m/z 102, m/z 295//m/z 130 and m/z 323//m/z 130 from dissociations of m/z 398 (Fig. 3C), m/z 426 (fig. S1C) and m/z 454 (Supplemntary Fig. 3B), respectively *via* molecular isomerization into ion-dipoles (i.e., ID_5, Supplementary Fig. 4a) composed by deprotonated GABA (or Leu) partner and bis substituted unsaturated γ lactone. This one can directly decompose yielding m/z 102 from m/z 398 (or m/z 130 from 426 and m/z 454). Alternatively, prior to dissociation, the ID_5 complex may isomerize by proton transfer from the γ lactone neutral to amino-acid. This modified ion-neutral then, fragments to give rise to formation of deprotonated bis substituted furan anion at m/z 295 from m/z 398 and from m/z 426 (or at m/z 323 from 454).

(b) m/z 198//m/z 199, m/z 198//m/z 227 and m/z 226//m/z 227 from dissociations of m/z 398 (Fig. 2C), m/z 426 (Supplementary Fig 1c), and m/z 454 (Supplementary Fig. 3B) which proceed *via* precursor ion isomerization into ion-neutrals (i.e., ID_6, Supplementary Fig. 4b). As previously, they can directly decompose into deprotonated fatty acid amides i.e., (i) m/z 198 from m/z 398 and m/z 426 and (ii) m/z 226 from m/z 454. Competitively, after internal proton transfer between both the partners of complex, they decompose leading to protonated deaminated dipeptides such as $[H_2NAsnGaba\mathit{OH}\text{-}H\text{-}NH_3]^-$ (m/z 199 from m/z 398) and $[H_2NAsnLeu\mathit{OH}\text{-}H\text{-}NH_3]^-$ (m/z 227 from 426 and m/z 454).

The mechanisms interpreting major fragmentations mainly involve formation of intermediates as ion-dipole complexes allowing often, by direct dissociation or through internal proton transfer, to give rise to formation of pairs of product ions whose is the sum of their respective m/z ratios correspond to the m/z value of molecular weight of the precursor anion.

Supplementary Table 1. High-resolution measurements by FT/MS of main product ions. Product ions were generated under HCD mode conditions (NCE = 35%) from deprotonated molecules prepared from the various studied lipoamino acids. All ions are represented in different fragmentation spectra represented in supplementary figure S9 where product ions were generated under HCD mode conditions at NCE = 20% or NCE=35%.

Selected precursor ions from the ESI ion source	Product ions			
	m/z values (experimental)	m/z values (calculated.)	Elemental composition	Formal loss
**341.2448**	323.2335	323.2335	$C_{18}H_{31}N_2O_3$	H_2O
	297.2532	297.2536	$C_{17}H_{33}N_2O_2$	CO_2
	279.2436	279.2431	$C_{17}H_{30}N_2O$	CH_2O_3
	252.2334	252.2327	$C_{16}H_{30}NO$	$C_2H_3NO_3$
	226.2176	226.2176	$C_{14}H_{28}NO$	$C_4H_5N_2O_3$
	131.0455	131.0456	$C_4H_7N_2O_3$	$C_{14}H_{26}O$
	114.0189	114.0191	$C_4H_4NO_3$	$C_{14}H_{29}NO$
	113.0353	113.0351	$C_4H_5N_2O_2$	$C_{14}H_{28}O_2$
	96.0078	96.0086	$C_4H_2NO_2$	$C_{14}H_{31}NO_2$
	89.0242	89.0239	$C_3H_5O_3$	$C_{15}H_{28}N_3O$
	87.0562	87.0558	$C_3H_7N_2O$	$C_{15}H_{26}O_3$
	70.0295	70.0293	C_3H_4NO	$C_{15}H_{29}NO_3$
	58.0296	58.0293	C_2H_4NO	$C_{16}H_{29}NO_3$
**398.2664**	312.2276[a]	312.2287	$C_{16}H_{30}N_3O_3$	$C_4H_6O_2$
	295.2029	295.2027	$C_{16}H_{27}N_2O_3$	$C_4H_9NO_2$
	224.2018	224.2014	$C_{14}H_{26}NO$	$C_6H_{10}N_2O_4$
	199.0722	199.0719	$C_8H_{11}N_2O_4$	$C_{12}H_{25}NO$
	198.1859	198.1858	$C_{12}H_{24}NO$	$C_8H_{12}N_2O_2$
	182.0454	182.0453	$C_8H_8NO_4$	$C_{12}H_{28}N_2O$
	155.0822[b]	155.0821	$C_7H_{11}N_2O_2$	$C_{13}H_{25}NO_3$
	138.0557 [b]	138.0556	$C_7H_8NO_2$	$C_{13}H_{28}N_2O_3$
	137.0716 [b]	137.0715	$C_7H_9N_2O$	$C_{13}H_{27}NO_4$
	113.0352[c]	113.0351	$C_4H_5N_2O_2$	$C_{16}H_{31}NO_3$
	102.0553[d]	102.0555	$C_4H_8NO_2$	$C_{16}H_{28}N_2O_3$
	98.0246[e]	98.0242	$C_4H_4NO_2$	$C_{16}H_{32}N_2O_3$
	96.0090[f]	96.0086	$C_4H_2NO_2$	$C_{16}H_{34}N_2O_3$
**313.2135**	295.2029	295.2027	$C_{16}H_{27}N_2O_3$	H_2O
	198.1859	198.1858	$C_{12}H_{24}NO$	$C_4H_5NO_3$
	131.0455	131.0456	$C_4H_7N_2O_3$	$C_{12}H_{22}O$
	114.0189	114.0191	$C_4H_4NO_3$	$C_{12}H_{25}NO$
	96.008	96.0086	$C_4H_2NO_2$	$C_{12}H_{27}N_2O_2$
**426.2980**	295.2029	295.2027	$C_{16}H_{27}N_2O_3$	$C_6H_{14}NO_2$
	227.1035	227.1032	$C_{10}H_{15}N_2O_4$	$C_{12}H_{26}NO$
	198.1860	198.1858	$C_{12}H_{24}NO$	$C_{10}H_{17}N_2O_4$
	130.0867	130.0868	$C_6H_{12}NO_2$	$C_{16}H_{29}N_2O_3$
	113.0349	113.0603	$C_6H_9O_2$	$C_{16}H_{32}N_3O_2$
	112.0760	112.0762	$C_6H_{10}NO$	$C_{16}H_{31}N_2O_4$

[a] Ion fragment pecific for BABA; [b] Ion fragment specific for GABA and AABA; [c] Most abundant fragment ion at NCE>35% for BABA; [d] Most abundant fragment ion at NCE>35% for AABA
[e] Ion fragment specific for AABA; [f] Most abundant fragment ion at NCE>35% for GABA

Supplementary Table 2. Strains and primers used in this study.

E. coli **strain**		**Reference**
**MG1655**	Serotype OR:H48:K-	Blattner *et al*;1997[3]
**MG1655+BAC *pks+***	MG1655 carrying BAC pks+,cm^r	Martin *et al.*,2013[4]
**Nissle 1917**	Serotype O6:K5:H1	Olier*et al.*, 2012[5]
**Nissle 1917 Δ*clbA***	*clbA*mutant strain, kan^r	Olier*et al.*, 2012[5]
**Nissle 1917 Δ*clbN***	*clbN*mutant strain, kan^r	this study
**Nissle 1917 Δ*clbB***	*clbB*mutant strain, kan^r	this study
**Nissle 1917 Δ*clbC***	*clbC*mutant strain, cm^r	this study
**Nissle 1917 Δ*clbP***	*clbP*mutant strain, kan^r	this study

**Primers**		
**clbN-P1**	cggtggcggtgattgatgatgtgcaggggcagcacagcg ggttgctagccgtgtaggctggagctgcttc	this study
**clbN-P2**	gacagtgctgacggcgtccagcgtgctgaggattaaatac caagagtgcacatatgaatatcctccttag	this study
**clbN-F**	cggtgattgatgatgtgcag	this study
**clbN-R**	gctgaggattaaataccaag	this study
**clbB-P1**	acatgccggtggcgattgtcggtatggcgggacgtttcccc ggtgcggcgtgtaggctggagctgcttc	this study
**clbB-P2**	tcgctgaagggtcgtcctcatgttgatgtgcttctagtgcgat gctggccatatgaatatcctccttag	this study
**clbB-F**	caacgcgtgctgttgtctac	this study
**clbB-R**	ctgaagggtcgtcctcatgt	this study
**clbC-P1**	gaaaggtaatgaagtttatggaatacgcaagcgaaatgaac ggcatggaagtgtaggctggagctgcttc	this study
**clbC-P2**	gggataaacaccggtgctcgcacttggtcatggtcatcgcg caccgcaggcatatgaatatcctccttag	this study
**clbC-F**	atatctcgaggtgaatacgcaagcgaaatgaac	this study
**clbC-R**	atataagcttttactgacaggctatttcgagg	this study
**clbP-P1**	ttccgctatgtgcgctttggcgcaagaacatgagcctatcgg ggcgcaagtgtaggctggagctgcttc	this study
**clbP-P2**	gtatacccggtgcgacatagagcatggcggccacgagcc caggaaccgcccatatgaatatcctccttag	this study
**ihapjpn29**	gtgaactgagcgaaatattggctaatc	Nougayrède et al., 2006[6]
**ihapjpn30**	ttactcatcgtcccactccttgttg	Nougayrède et al., 2006[6]

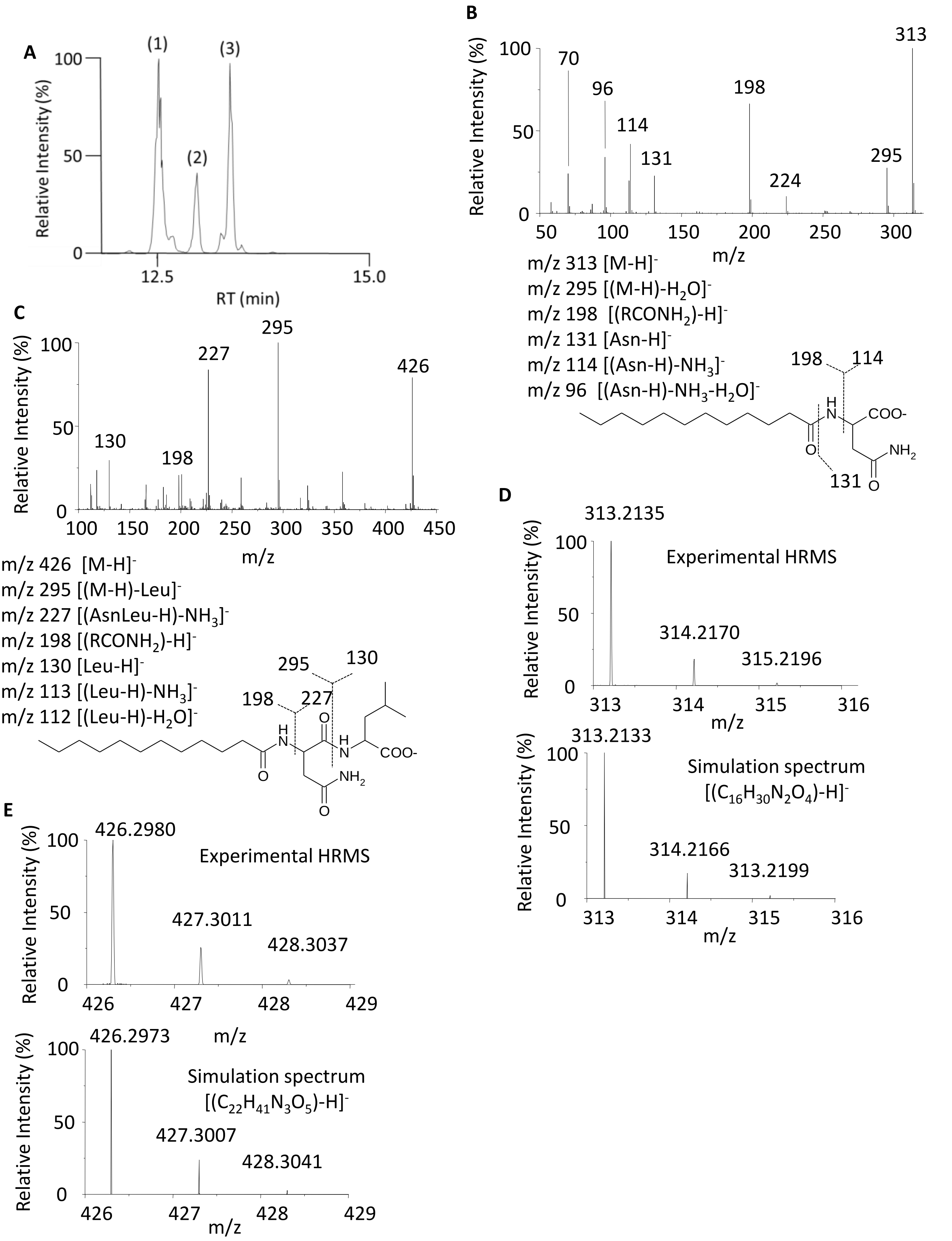

Supplementary Figure 1: Characterization of C12-Asparagine (C12-Asn-OH) and C12-Asparagine-Leucine (C12-Asn-Leu-OH) by LC-HRMS. (**A**) Extracted Ion Chromatogram (EIC), the common m/z 295.2021 product ion from dissociation of both the [(C12Asn*OH*)-H]$^{-}$and

[(C12AsnLeu*OH*)-H]$^-$anions in a lipidic extract of probiotic bacteria pellet. Peak (1) is related to C12-Asn-OH, peak (2) is C12-Asn-ABA and peak (3) is C12-Asn-Leu-OH. (**B**) Product ion spectrum *via* the HCD mode of the carboxylate [M-H]$^-$ anion (m/z 313) generated by electrospray from the LC peak (1) *i.e.*, C12-Asn-OH. (**C**) HCD spectrum of the carboxylate [M-H]$^-$anion (m/z 426) formed in electrospray from the LC peak (3) *i.e.*, C12-Asn-Leu-OH. (**D**) Natural isotopic distribution of the deprotonated molecule displayed by the high resolution mass spectrum zoom obtained for the peak (1) in the probiotic strain EIC (top) and natural isotopic pattern calculated with the formula [($C_{16}H_{30}N_2O_4$)-H]$^-$. Analogous natural isotopic patterns and similar m/z ratios measured and simulated for the mono-isotopic [($^{12}C_{16}{}^{1}H_{30}{}^{14}N_2{}^{16}O_4$)-H]$^-$ ion and for the [($^{13}C_nC_{16-n}{}^{1}H_{30}{}^{14}N_2{}^{16}O_4$)-H]$^-$ (with n+1 and 2) ions (within an accuracy of 0.6 ppm).(**E**) Natural isotopic distribution of the deprotonated molecule displayed by the high resolution mass spectrum zoom obtained for the peak (3) in the probiotic strain EIC (top) and simulated for the mono-isotopic [($^{12}C_{22}{}^{1}H_{41}{}^{14}N_3{}^{16}O_5$)-H]$^-$ion and for the [($^{13}C_nC_{22-n}{}^{1}H_{41}{}^{14}N_3{}^{16}O_5$)-H]$^-$(with n+1 and 2) ions (within an accuracy of 1.6 ppm).

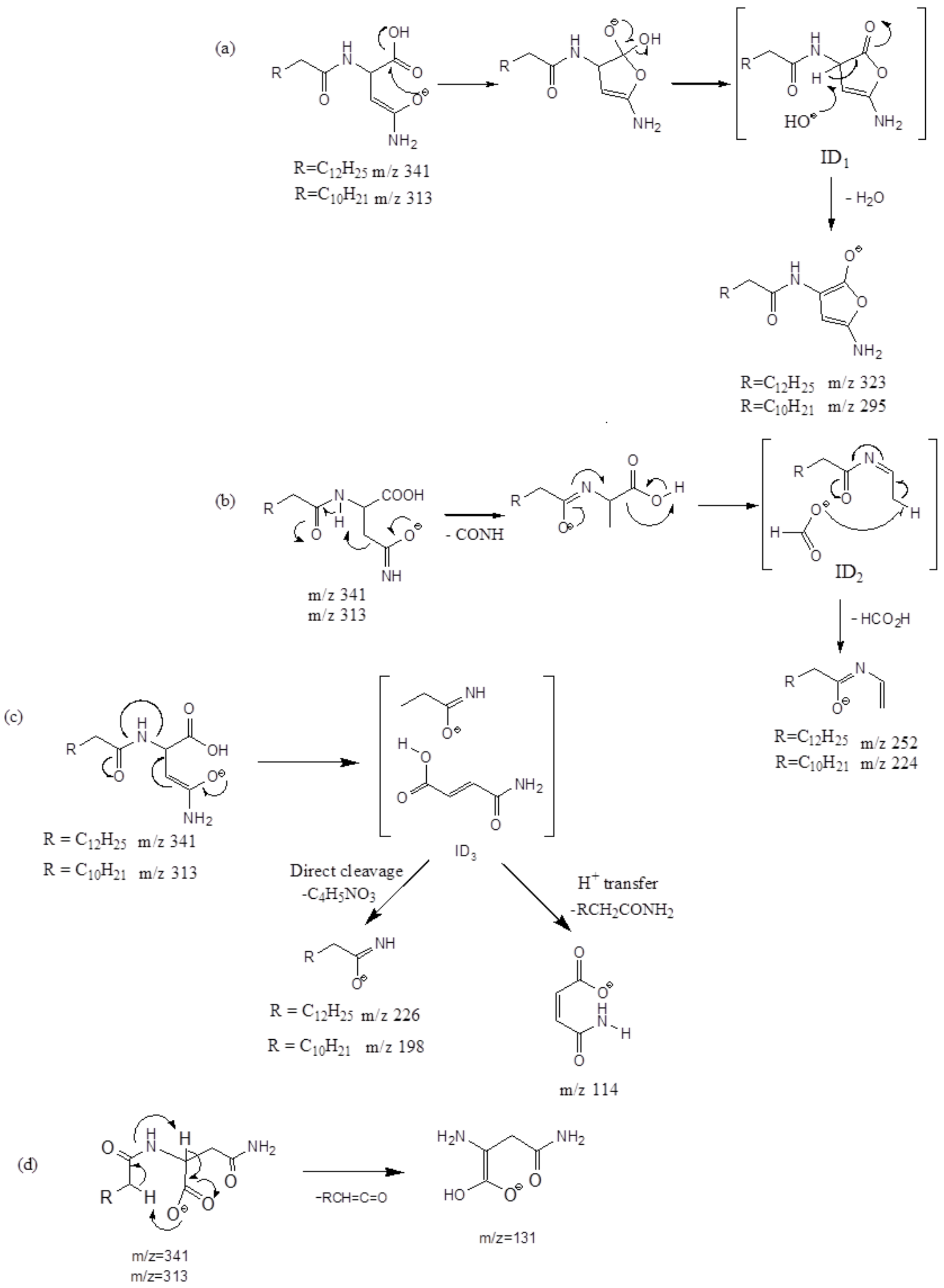

Supplementary Figure 2: Proposed mechanisms to interpret formation of product ions of the first generation under HCD conditions from $[M\text{-}H]^-$ (m/z 341 and m/z 323) as $[RCH_2CONHCH(COOH)CH_2CONH_2\text{-}H]^-$ (with $R=CH_3(CH_2)_{11}^-$ and $CH_3(CH_2)_9^-$ displayed in figures 2C and Supplementary 1b)

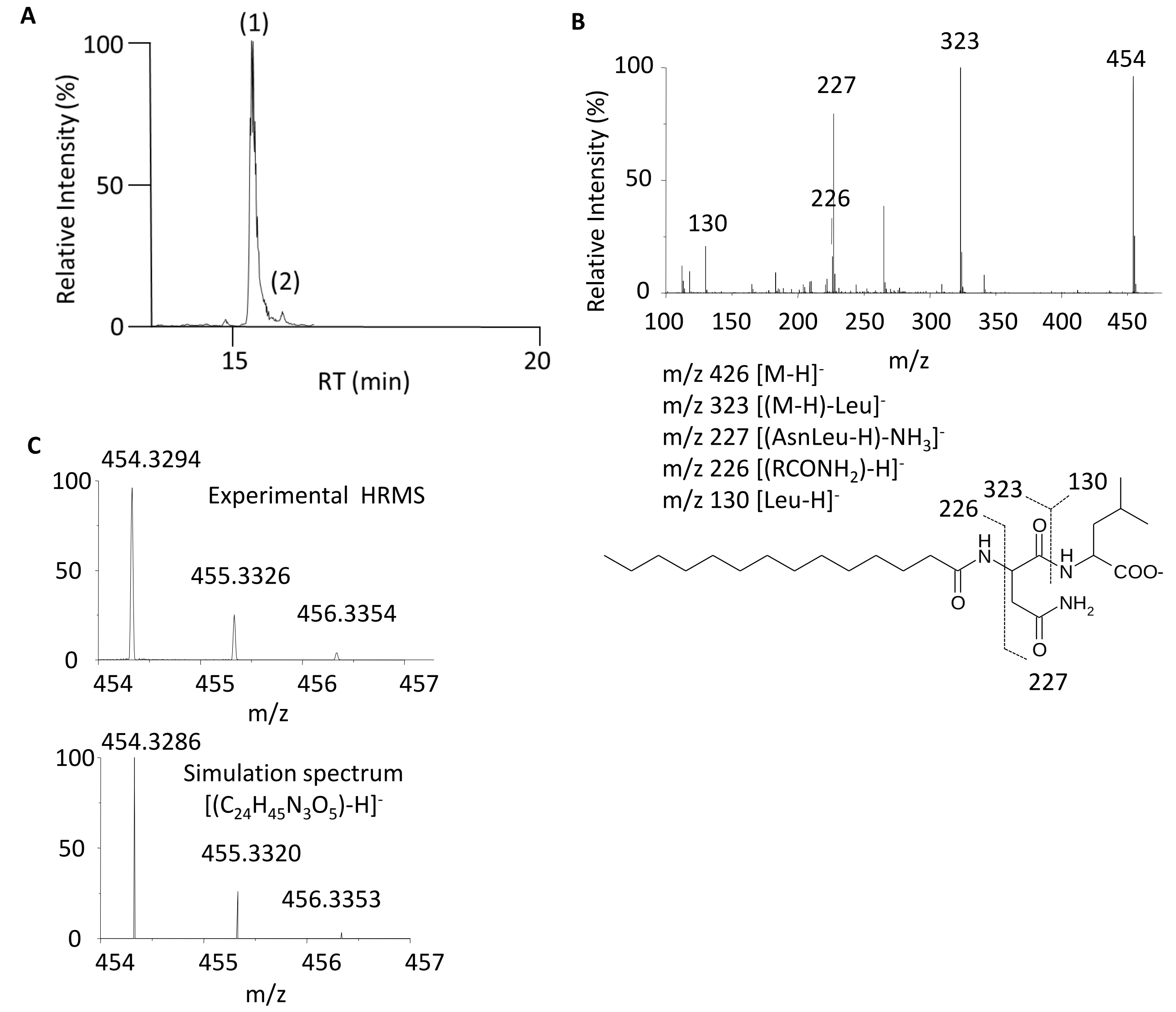

Supplementary Figure 3: Characterization of C14-Asparagine-Leucine (C14-Asn-Leu-OH) by LC-HRMS. (**A**) Extracted Ion Chromatogram (EIC) of the m/z323.2335 product ion corresponding to [(C14Asn*OH*)-H-H_2O]$^-$ as by leucine release from the [(C14AsnLeu*OH*)-H]$^-$anion generated in electrospray from a lipid extract of probiotic bacteria pellet. Peak (1) is C14-Asn-OH and peak (2) is C14-Asn-Leu-OH. (**B**) Product ion spectrum acquired in HCD mode of the carboxylate [M-H]$^-$ anion prepared in electrospray from the LC peak (2) *i.e.,* C12-Asn-Leu-OH. (**C**) Natural isotopic distribution of the deprotonated molecule m/z 454.3284 displayed by the high resolution mass spectrum zoom obtained for the peak (2) in the probiotic strain EIC (top) similar to that of simulated profile for the mono-isotopic [($^{12}C_{24}{}^{1}H_{45}{}^{14}N_{3}{}^{16}O_{5}$)-H]$^-$ ion and for the [($^{13}C_{n}C_{24-n}{}^{1}H_{45}{}^{14}N_{3}{}^{16}O_{5}$)-H]$^-$ (with n+1 and 2) ions (within an accuracy of 1.8 ppm).

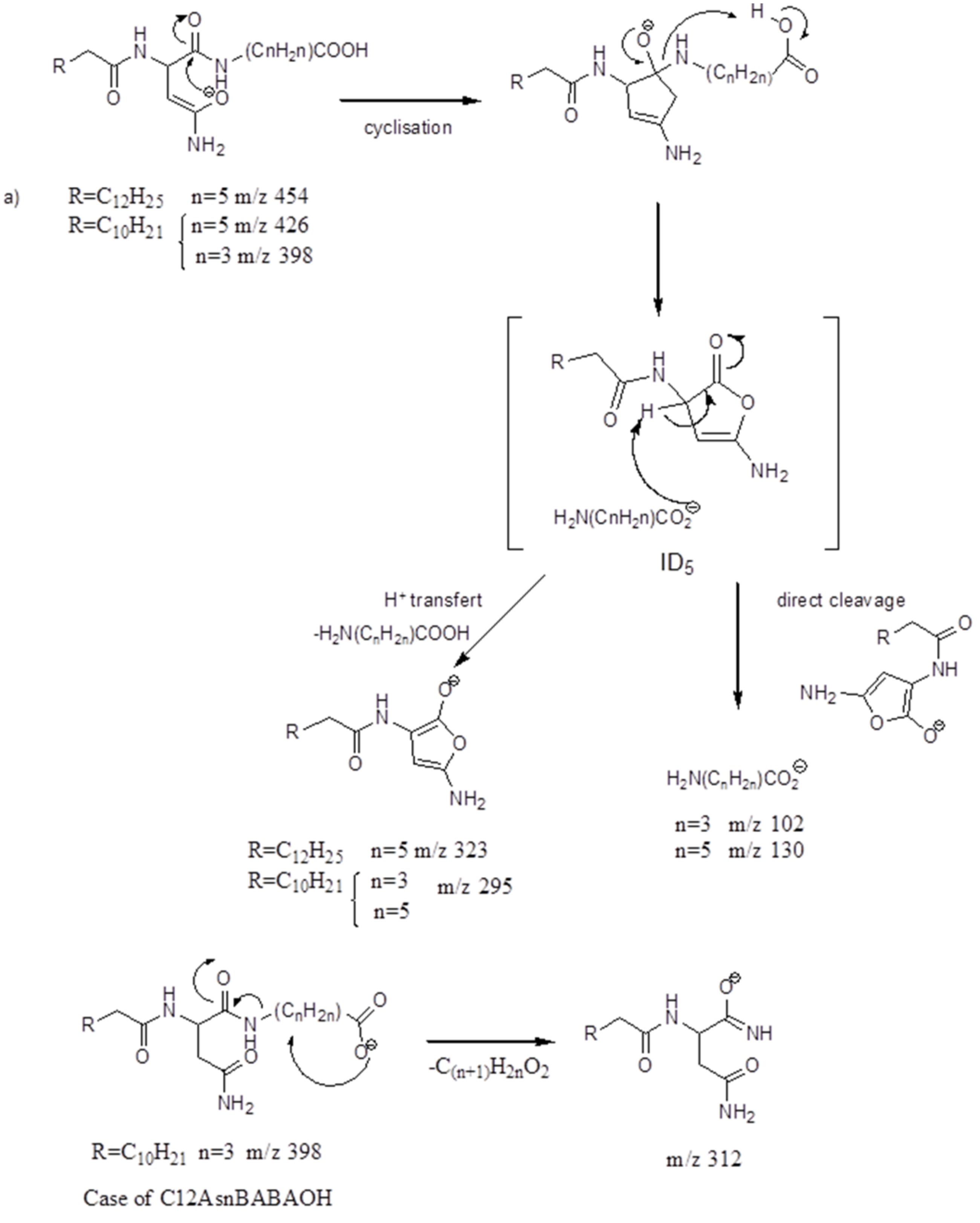

cyclisation
a) R=C12H25 n=5 m/z 454
R=C10H21 n=5 m/z 426
n=3 m/z 398
H2N(CnH2n)CO2
ID5
H+ transfert
-H2N(CnH2n)COOH
direct cleavage
R=C12H25 n=5 m/z 323
R=C10H21 n=3 n=5 m/z 295
H2N(CnH2n)CO2
n=3 m/z 102
n=5 m/z 130
-C(n+1)H2nO2
R=C10H21 n=3 m/z 398
Case of C12AsnBABAOH
m/z 312

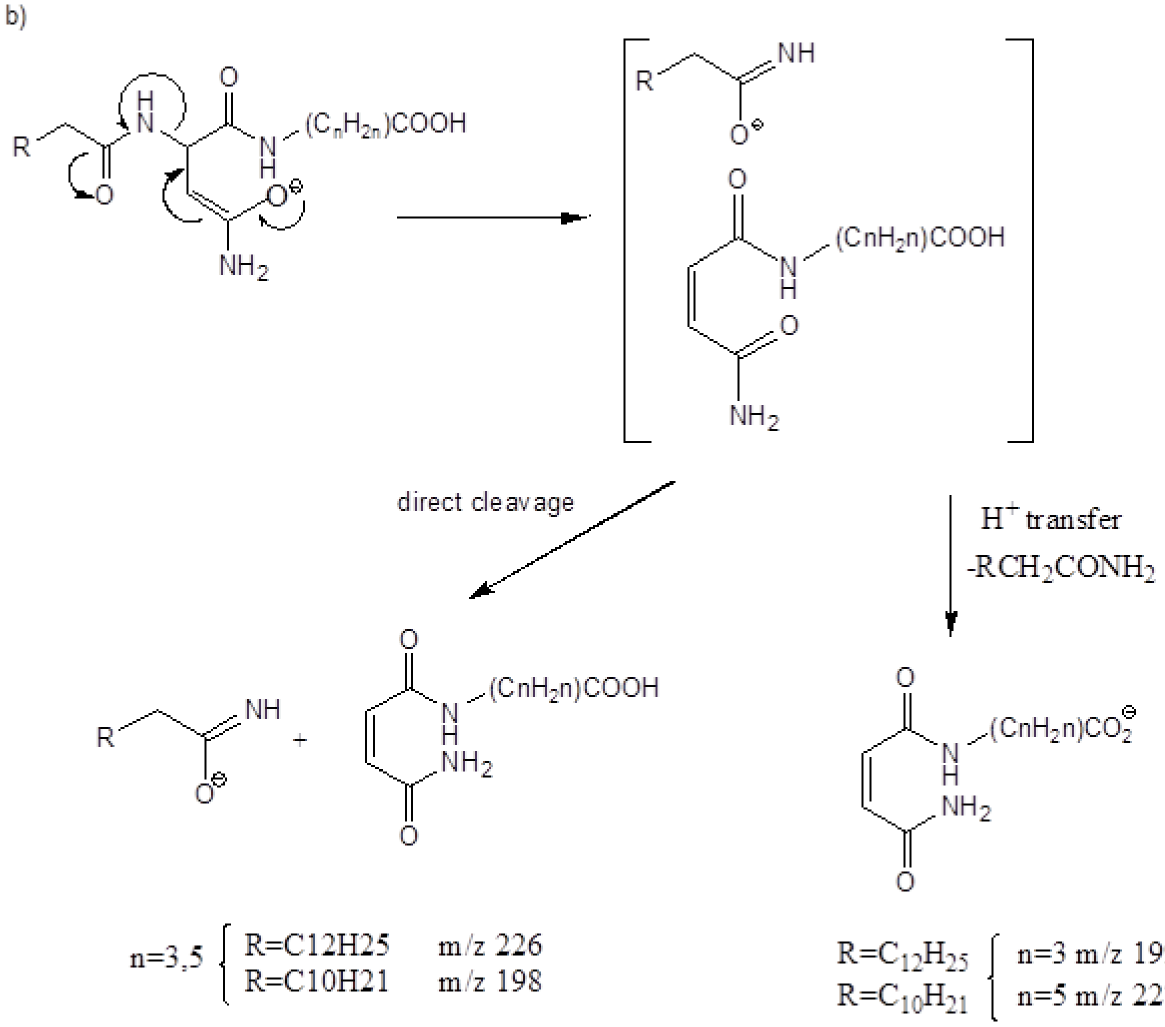

Supplementary Figure 4: Proposed interpretation of major common product ions generated under HCD conditions from the $[M-H]^-$ precursor ions (m/z 398; m/z 426 and m/z 454 as $RCH_2CONHCH(CH_2CONH_2)CONH-(C_nH_{2n})COOH$ with (i) $R=C_{12}H_{25}$ and $C_{10}H_{21}$ and (ii) n=3 (GABA, BABA, AABA) and n=5 (leucine or isoleucine)

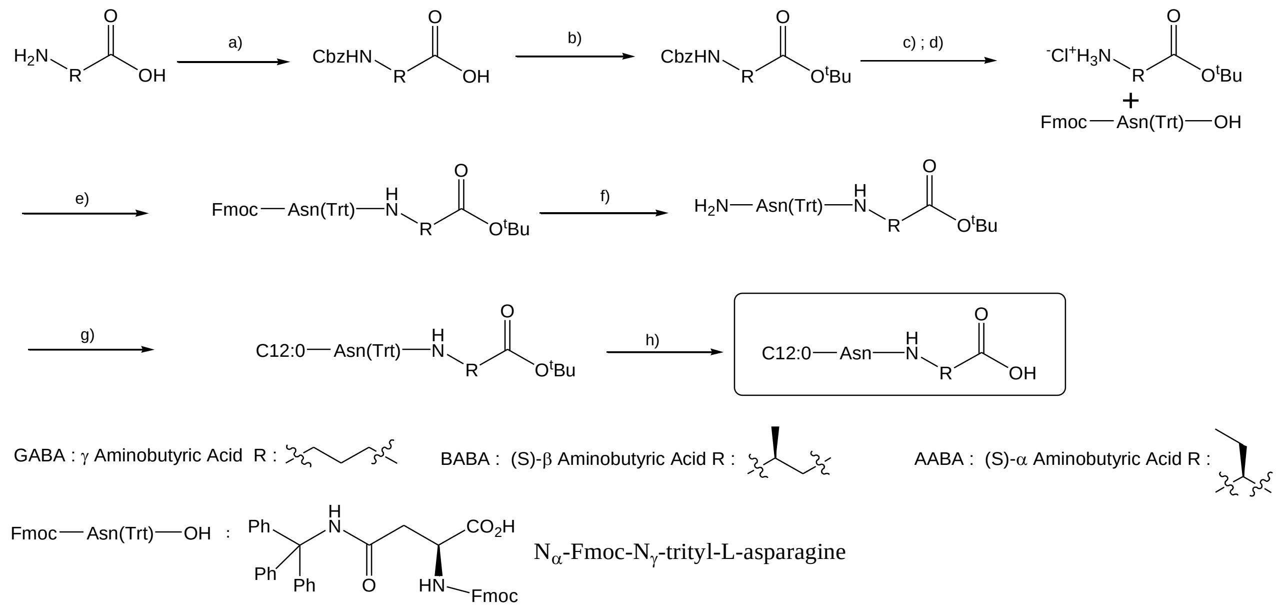

Supplementary Figure 5: Synthesis of C12-Asn-AABA*OH*, -BABA*OH* and -GABA*OH*. a) Cbz-Cl, NaOH, 0 °C; b) DCC, DMAP, ^{t}BuOH, 36% to 50%; c) H_2, Pd/C, MeOH; d) HCl, Et_2O, 50% to 82%; e) HBTU, HOBt, NMM, CH_2Cl_2, 90% to 97%; f) Et_2NH, CH_2Cl_2; g) HBTU, HOBt, NMM, Lauric Acid CH_2Cl_2, 59% to 70%; h) TFA, CH_2Cl_2, HPLC purification, 8% to 15%.***Abreviations:*** *AABA : L-α-Aminobutyric Acid, BABA : (S)-β- Aminobutyric Acid, GABA : γ-Aminobutyric Acid, Cbz-Cl : Benzyl Chloroformate, DCC :Dicyclohexylcarbodiimide, DMAP : 4-(Dimethylamino)pyridine, HBTU : N,N,N',N'-Tetramethyl-O-(1H-benzotriazol-1-yl) uroniumhexafluorophosphate, HOBt : 1-Hydroxybenzotriazole hydrate, NMM : 4-Methylmorpholine, TFA : Trifluoroacetic Acid.*

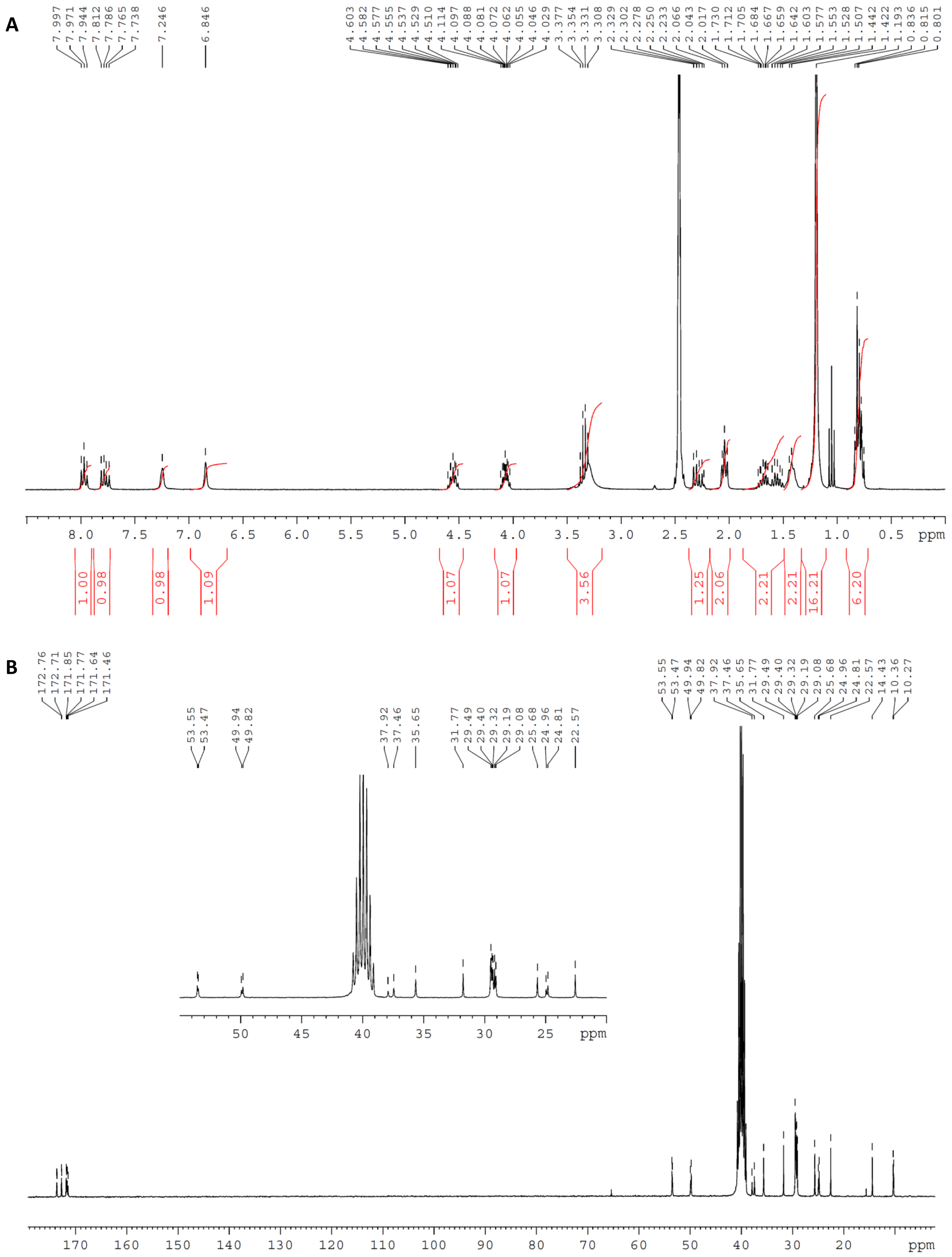

Supplementary Figure 6: ^{1}H-NMR spectrum (**A**) and ^{13}C-NMR spectrum (**B**) of C12AsnAABA in DMSO.

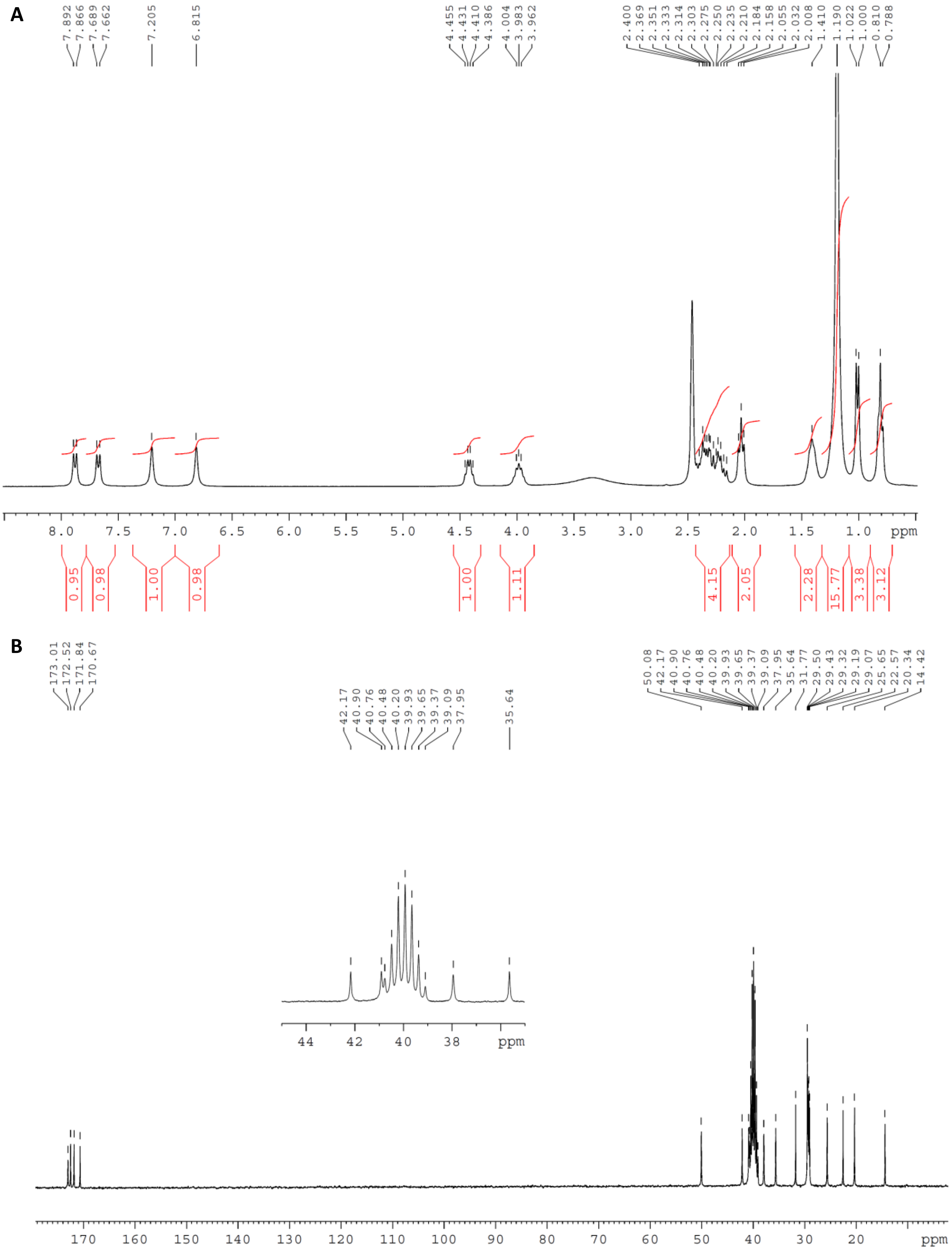

Supplementary Figure 7: ^{1}H-NMR spectrum (**A**) and ^{13}C-NMR spectrum (**B**) of C12AsnBABA in DMSO.

A

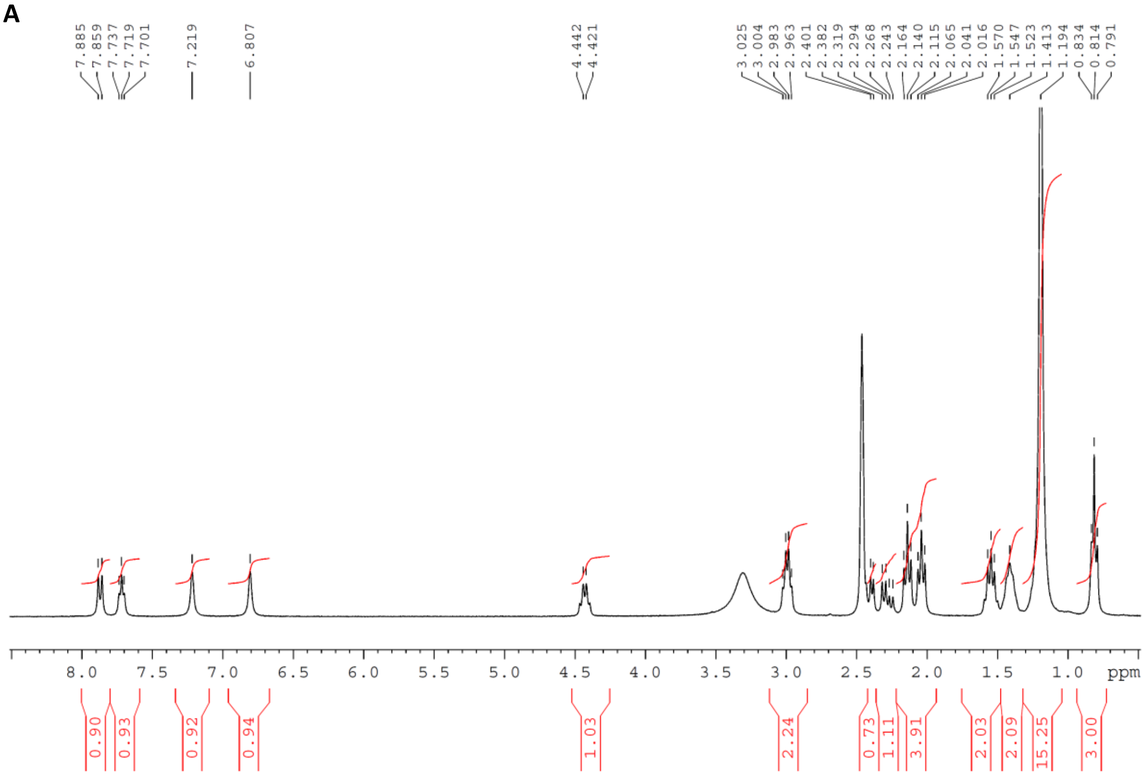

B

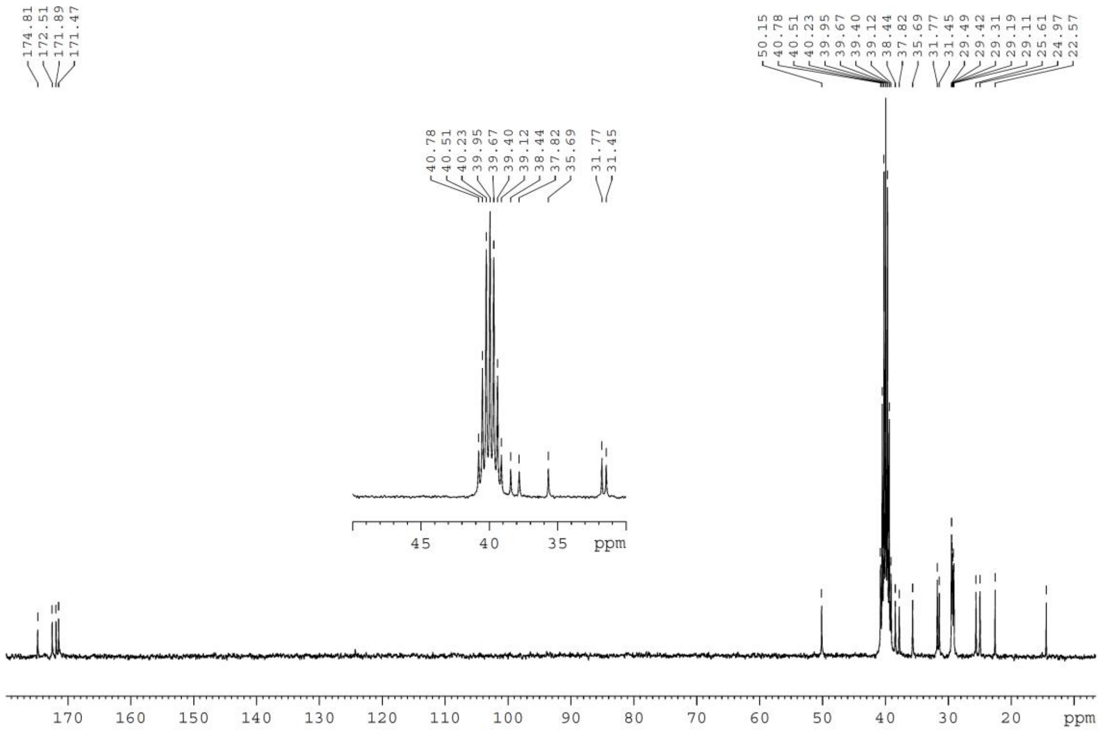

Supplementary Figure 8: ^{1}H-NMR spectrum (**A**) and ^{13}C-NMR spectrum (**B**) of C12AsnGABA in DMSO.

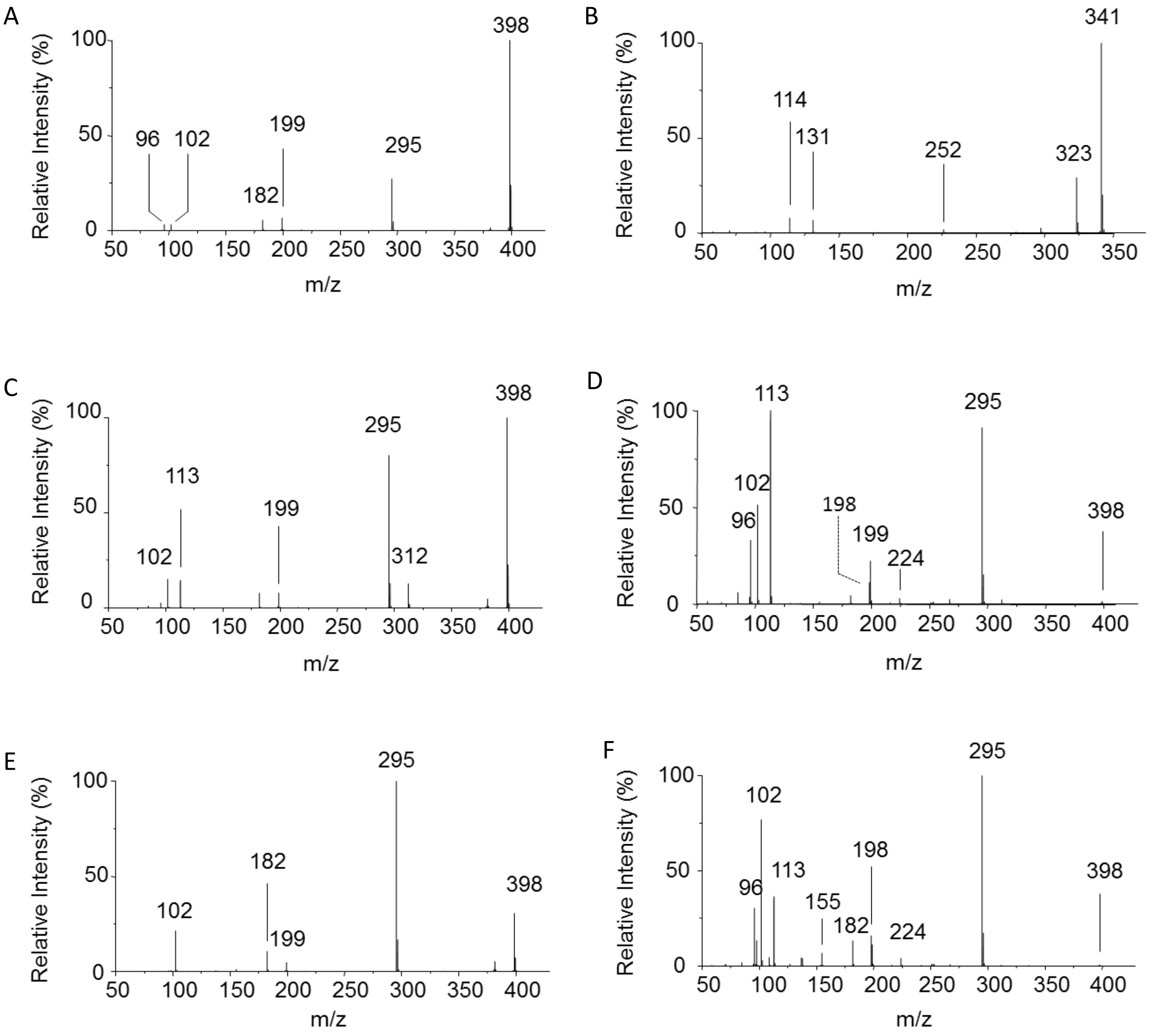

Supplementary Figure 9: Product ion spectra acquired in HCD mode of isomeric deprotonated molecules on Q exactive+ mass spectrometer: (A) product ion spectrum of C12AsnGABA*OH* at NCE 20% (experiment at NCE 35 % reported in Fig 2C); (B) product ion spectrum of C14Asn*OH* at NCE 20% (that of analog C12Asn*OH* at NCE 35 % reported in Fig 1C); (C) product ion spectrum of C12AsnBABA*OH* at NCE 20%; (D) product ion spectrum of C12AsnBABA*OH* at NCE 35%; (E) product ion spectrum of C12AsnAABA*OH at* NCE 20%; (F) and product ion spectrum of C12AsnAABA*OH* at NCE 35%.

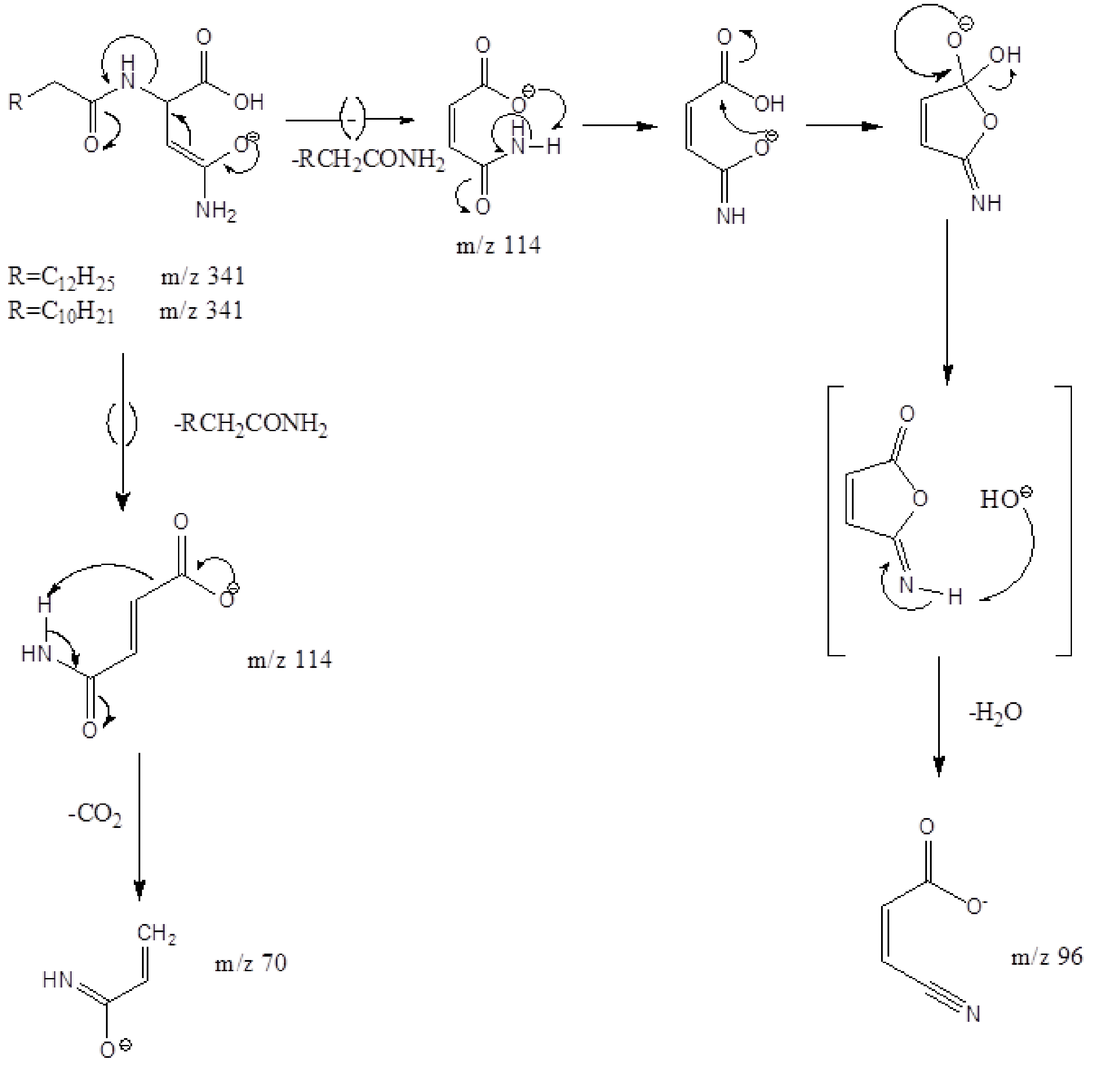

Supplementary Figure 10: Possible interpretation of product ions displayed at low m/z range (*ie*., from m/z 50 to m/z 135) in HCD spectra of $C_{12/14}$Asn*OH*

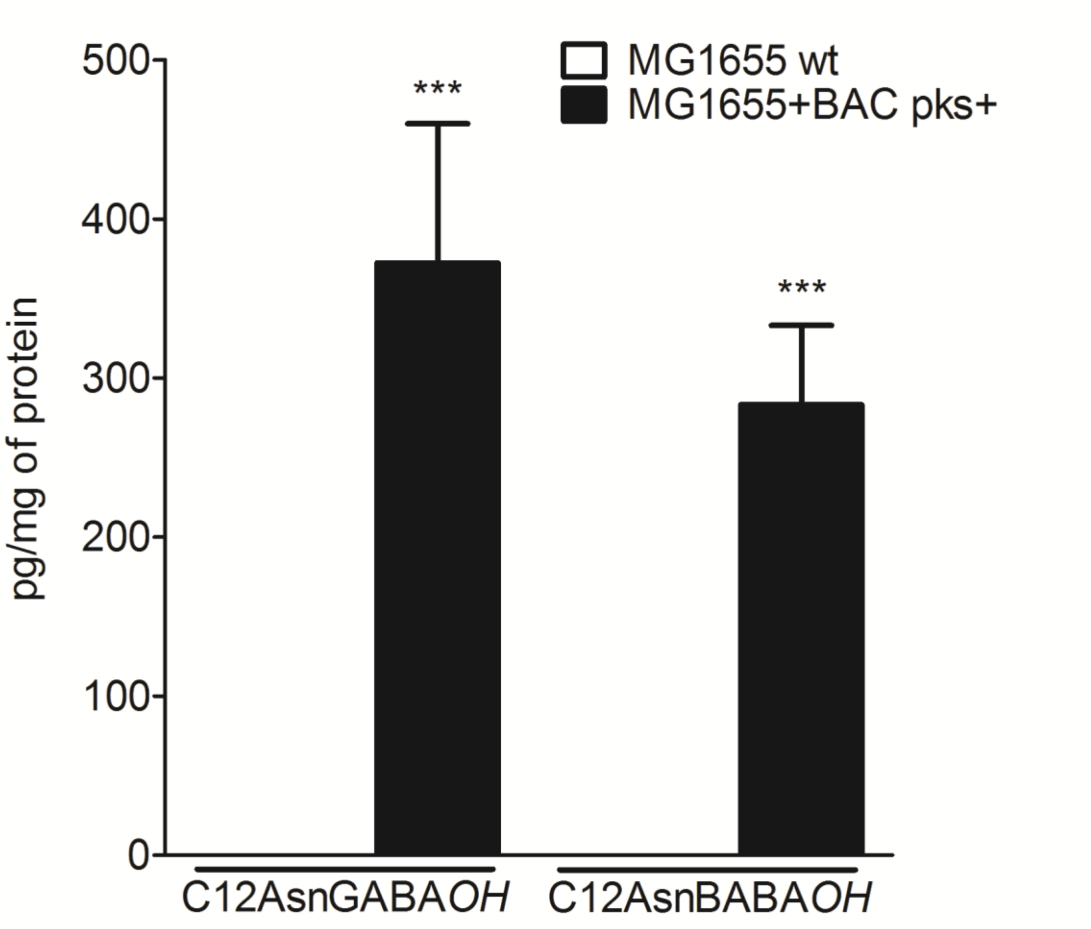

Supplementary Figure 11: Quantification of C12AsnGABA*OH* and C12AsnBABA*OH*. C12AsnGABA*OH* and C12AsnBABA*OH* were quantified by LC-MS/MS in pellets of MG1655 wild-type and MG1655+BAC pks+. Data are represented as mean ± SEM of 3 experiments of 2 independent bacterial cultures per group. Statistical analysis was performed using Kruskal-Wallis analysis of variance and subsequent Dunn's post hoc test. *** $p < 0.001$, significantly different from MG1655 wt.

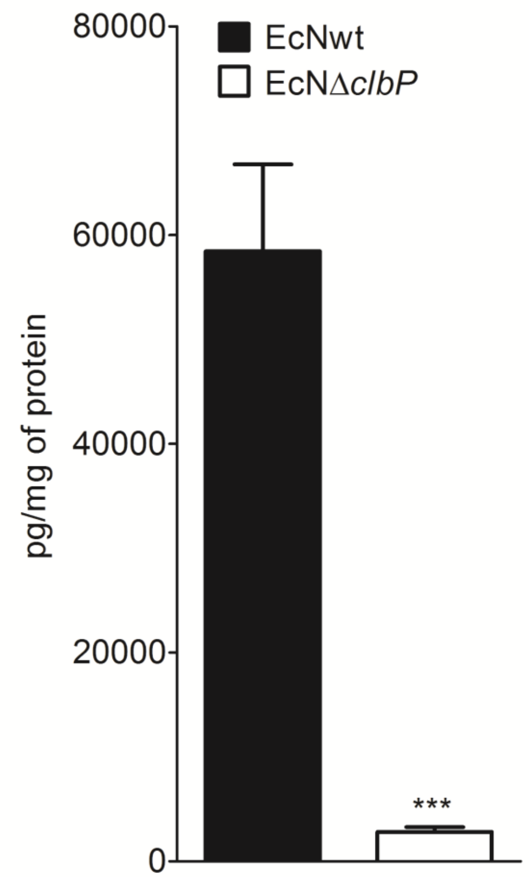

Supplementary Figure 12: quantification of C14-Asparagine by LC-QQQ. Synthesis of C14Asn*OH* was performed as described for the synthesis of C12AsnGABA*OH* and C12AsnBABA*OH*. Quantification of C14Asn*OH* was performed as described for C12AsnGABA*OH* and C12AsnBABA*OH* in pellets of EcNwt and EcNΔ*clbP*. Data are represented as mean ± SEM of 2 experiments of 6 independent bacterial cultures per group. Statistical analysis was performed using Mann-Whitney test. *** $p < 0.001$, significantly different from EcNwt.

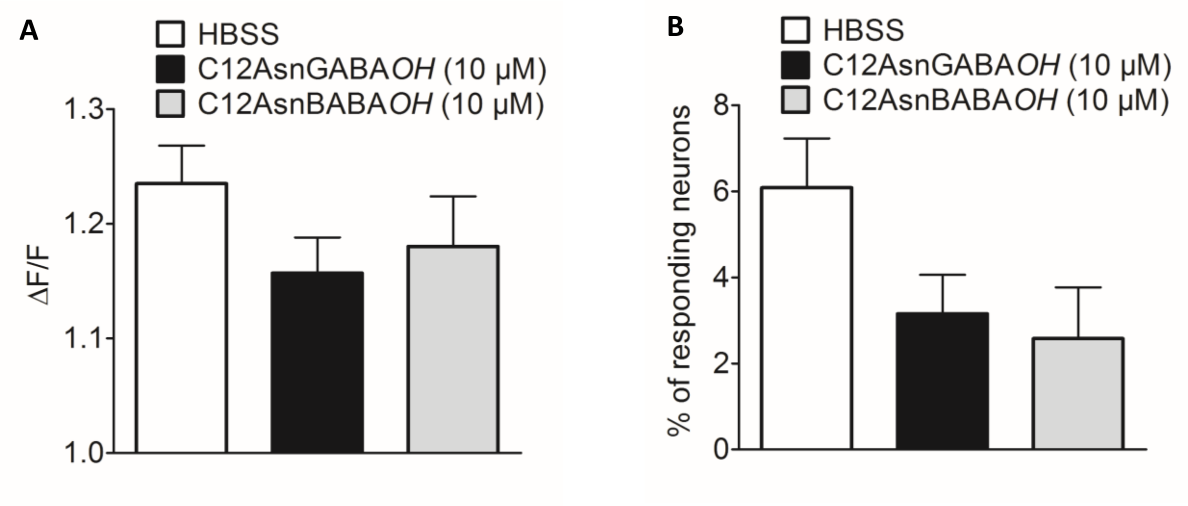

Supplementary Figure 13: C12AsnGABA*OH* and C12AsnBABA*OH* did not induce calcium flux in sensory neurons. Amplitude of intracellular calcium mobilization (ΔF/F; **A**) in mouse sensory neurons and percentage of responding neurons (**B)** treated with 10 µM of C12AsnGABA*OH* (black bars), C12AsnBABA*OH* (gray bars) or vehicle (HBSS; white bars). Data are represented as mean ± SEM; n=3 independent experiments of 3 wells per condition and 30-80 neurons per well. Statistical analysis was performed using Kruskal-Wallis analysis of variance and subsequent Dunn's post hoc test.

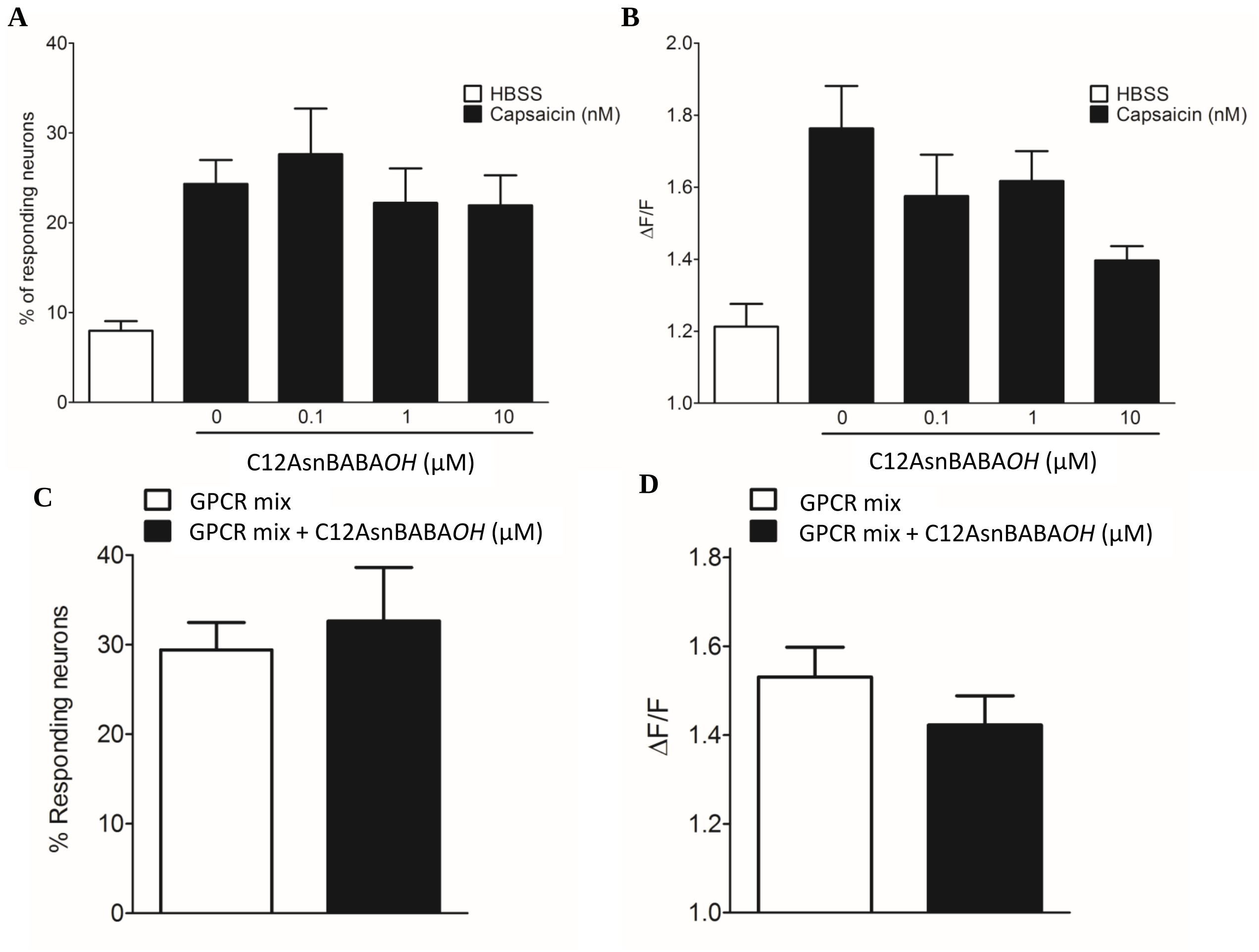

Supplementary Figure 14: C12AsnBABA*OH* does not decrease neuronal activation. Amplitude of intracellular calcium mobilization (ΔF/F; A and C) in mouse sensory neurons and percentage of responding neurons (B and D) pretreated with C12AsnBABA*OH* (black bars) or vehicle (HBSS; white bars) and treated with capsaicin (125 nM; A and B) or a mix of G protein coupled receptor agonist (histamine, serotonin and bradykinin 10 µM each; C and D). Data are represented as mean ± SEM; n=3 independent experiments of 3 wells per condition and 30-80 neurons per well. Statistical analysis was performed using Kruskal-Wallis analysis of variance and subsequent Dunn's post hoc test.

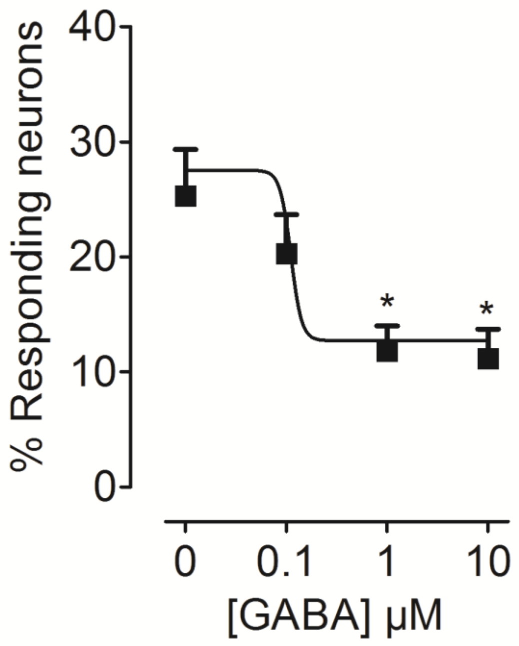

Supplementary Figure 15: GABA does decrease neuronal activation. Percentage of responding neurons from mouse dorsal root ganglia pretreated with different concentration of GABA and treated with capsaicin (125 nM). Data are represented as mean ± SEM; n=3 independent experiments of 3 wells per condition and 25-60 neurons per well. Statistical analysis was performed using Kruskal-Wallis analysis of variance and subsequent Dunn's post hoc test. * $p < 0.05$, significantly different from capsaicin alone.

Supplementary References

1. Afonso, C., Cole, R.B. & Tabet., J.C. (eds.). *Dissociation of Even-Electron Ions. Electrospray and MALDI Mass Spectrometry: Fundamentals, Instrumentation, Practicalities, and Biological Applications, Second Edition.*, 631-82 (2010).
2. Boukerche, T.T. et al. Atypical cleavage of protonated N-fatty acyl amino acids derived from aspartic acid evidenced by sequential MS3 experiments. *Amino Acids* **48**, 2717-2729 (2016).
3. Blattner, F.R. et al. The complete genome sequence of Escherichia coli K-12. *Science* **277**, 1453-62 (1997).
4. Martin, P. et al. Interplay between siderophores and colibactin genotoxin biosynthetic pathways in Escherichia coli. *PLoS Pathog* **9**, e1003437 (2013).
5. Olier, M. et al. Genotoxicity of Escherichia coli Nissle 1917 strain cannot be dissociated from its probiotic activity. *Gut Microbes* **3**, 501-9 (2012).
6. Nougayrede, J.P. et al. Escherichia coli induces DNA double-strand breaks in eukaryotic cells. *Science* **313**, 848-51 (2006).
